# Subannular repair for functional mitral regurgitation with reduced systolic ventricle function: rationale and design of REFORM-MR registry

**DOI:** 10.1186/s13019-022-02045-9

**Published:** 2022-12-30

**Authors:** Evaldas Girdauskas, Jonas Pausch, Hermann Reichenspurner, Jörg Kempfert, Thomas Kuntze, Tamer Owais, Tomas Holubec, Markus Krane, Keti Vitanova, Michael Borger, Matthias Eden, Violetta Hachaturyan, Peter Bramlage, Volkmar Falk

**Affiliations:** 1 Department of Cardiovascular Surgery, University Heart and Vascular Center Hamburg, Hamburg, Germany; 2 Department of Cardiovascular and Thoracic Surgery, University Hospital Augsburg, Augsburg, Germany; 3 Department of Cardiothoracic and Vascular Surgery, German Heart Center Berlin, Berlin, Germany; 4 German Center for Cardiovascular Research, Partner Site Berlin, Berlin, Germany; 5 Department of Cardiology, Central Hospital Bad Berka, Bad Berka, Germany; 6 Department of Cardiovascular Surgery, University Hospital Frankfurt, Johann Wolfgang Goethe University Frankfurt, Frankfurt am Main, Germany; 7 German Heart Center Munich, Munich, Germany; 8 Department Cardiac Surgery, Leipzig Heart Center, University Clinic Leipzig, Leipzig, Germany; 9 Department for Internal Medicine III, Molecular Cardiology and Angiology, University Hospital Schleswig-Holstein, Kiel, Germany; 10 Institute for Pharmacology and Preventive Medicine, Bahnhofstrasse 20, 49661 Cloppenburg, Germany; 11 Department of Cardiovascular Surgery, Charité—Universitätsmedizin Berlin, Corporate Member of Freie Universität Berlin, Humboldt Universität zu Berlin, Berlin, Germany; 12 Department of Health Sciences and Technology, ETH Zürich, Zurich, Switzerland

**Keywords:** Functional mitral regurgitation, Mitral regurgitation, Mitral valve repair, Mitral annuloplasty, Subannular repair

## Abstract

**Background:**

Functional mitral regurgitation (FMR) is one of the most common heart valve diseases that is a sequel of left ventricular remodelling. Although mitral valve annuloplasty is a standard treatment of FMR, the recurrence of FMR is a major drawback and occurs in 10–50% of patients. The REFORM-MR registry aims to investigate the effectiveness of standardized papillary muscle relocation and ring annuloplasty and to identify the risk factors associated with recurrent FMR.

**Methods:**

REFORM-MR is a prospective, multicenter registry that enrols consecutive FMR patients across five sites in Germany. All patients with FMR and restricted movement of leaflets during systole (i.e., type IIIb mitral regurgitation) undergoing standardized subannular repair in combination with mitral valve annuloplasty are included in the study. The primary objective is to examine the effect of combined papillary muscle relocation and ring annuloplasty on the recurrence of FMR at 2 years postoperatively. The secondary objectives are MACCE rate, reinterventions on the mitral valve and cardiac-related mortality in the study cohort. Echocardiography core-lab and MRI core-lab will provide anonymized analysis of the imaging data in the REFORM-MR registry. Student’s *t*-test or Mann–Whitney *U* test for continuous variables and the Chi-Square or Fisher exact test for categorical variables are used for group comparisons. Kaplan–Meier analyses is performed for survival and safety outcomes.

**Results:**

As of May 2021, a total of 97 patients were enrolled across five sites in Germany.

**Conclusions:**

The results of this study will help define the outcomes of combined papillary muscle relocation and ring annuloplasty in the FMR treatment in a multicentre setting and to improve the understanding of the limitations of subannular repair procedures while treating patients with type III FMR.

*Trial registration* clinicaltrials.gov Identifier: NCT03470155.

## Background

Mitral regurgitation (MR) is one of the most common valvular diseases in adults that affects more than 2% of the general population worldwide and has an increasing prevalence with age [1]. Functional (or secondary) mitral regurgitation (FMR) results from annular dilation (i.e., type I MR) and/or apico-lateral papillary muscle displacement due to left ventricular (LV) remodelling (type IIIb MR). LV enlargement with the resulting geometric dislocation of the papillary muscles and increased distance between papillary muscle tips and mitral annular plane leads to restricted systolic motion of the mitral valve leaflets (so-called mitral valve tethering) [2]. FMR is associated with increased morbidity and mortality [3]. The treatment of FMR has significantly changed over the last decades, while mitral valve annuloplasty remains the most frequently used surgical technique. However, despite the fact that mitral valve annuloplasty has been shown to reduce the severity of FMR and improve the heart failure symptoms in the short term, it has also been associated with a high recurrence rate of FMR [4–6].

The recurrence rate of moderate to severe FMR in patients during follow-up after mitral valve annuloplasty has been reported in the range of 10–50%, resulting in an increased long-term mortality, new or worsening heart failure, and compromised quality of life (QoL) [5, 7, 8]. A number of studies examined technical and clinical factors that may reduce the risk of FMR recurrence after mitral valve repair (MVr), including the cause of FMR, type of the surgical procedure, anatomical characteristics of the mitral valve, and pre-existing medical conditions [5]. However, there is still an urgent clinical demand to identify reliable pre-procedural parameters that may predict MVr failure and FMR recurrence.

A subgroup of FMR patients presented with echocardiographic evidence of reverse LV remodelling after an isolated ring annuloplasty, while a continued LV remodelling was observed in the remaining FMR patients despite an initially successful mitral valve annuloplasty [9, 10]. In the case of progressive LV remodelling, recurrent FMR is mostly due to increasing leaflet tethering. This is because the ring annuloplasty primarily targets mitral valve annulus dilation and does not relieve leaflet tethering due to papillary muscle displacement. To address the tethering issue, several subannular repair techniques have been developed to ameliorate the drawbacks of isolated ring annuloplasty. Subannular techniques describe a wide spectrum of surgical manoeuvres, including cutting of the secondary chordae, patch augmentation of the posterior or anterior mitral leaflet, papillary muscle approximation, and papillary muscle relocation [11–14]. A recent meta-analysis has highlighted potential advantages of combining ring annuloplasty with a papillary muscle intervention to enable a complete geometric MVr and thereby to reduce the recurrence rate of FMR [15]. However, due to lack of robust data on FMR treatment from well-designed, prospective, multicenter trials, clinical adoption of papillary muscle techniques is still very limited.

We designed the prospective, multicentre REFORM-MR registry to assess the feasibility and efficacy of a combined subannular repair by relocation of papillary muscles and ring annuloplasty to reduce the risk of recurrent FMR in patients with type IIIb MR. In addition, we aimed to identify pre-procedural characteristics and echocardiographic parameters that may predict FMR recurrence after such procedure. Taken together, these findings could provide strong evidence for standardization and consistency in the clinical practice while treating patients with the type IIIb FMR.

## Methods/design

REFORM-MR is a prospective, observational, multicenter registry with a follow-up of 2 years to assess the effectiveness of standardized subannular repair by papillary muscle relocation and annuloplasty on the FMR recurrence, Major Adverse Cardiac and Cerebrovascular Events (MACCE), and QoL at 2 years following surgery. The registry was registered at www.clinicaltrials.gov, Identifier NCT03470155.

### Participating sites

Sites were selected based on recommendations of the principal investigator and included only high-volume surgical centers in Germany performing > 100 mitral valve surgery cases/year. The prerequisites for site’s participation were an extensive experience in mitral valve surgery, including minimally invasive techniques, and the site’s readiness in using the standardized subannular repair technique in consecutive FMR patients.

In preselected centers, the most experienced mitral surgeons were included and all of them had mitral valve case-load > 50 procedures/year over the last years. The training program in all participating centers included a theoretical teaching unit, visiting of the Core center with a watching of live-surgery, and an individual proctoring of the first 2–4 cases by the first author of  the manuscript.

### Patients

Patients were enrolled in the registry based on the following inclusion criteria: (1) presence of FMR with impaired movement of leaflets during systole (type IIIb)—effective regurgitation orifice area more than 20 mm^2^/regurgitant volume more than 30 ml/beat, (2) left ventricular ejection fraction less than 50%, (3) left ventricular end-diastolic diameter of at least 55 mm, and (4) tenting height of the posterior and/or anterior mitral leaflets > 10 mm (Table 1). Patients were excluded based on the exclusion criteria summarized in Table 1. Written informed consent was obtained from all patients prior to enrolment and compliance with the Declaration of Helsinki was ensured throughout the trial.

**Table 1:** REFORM-MR inclusion/exclusion criteria

Inclusion criteria	Exclusion criteria
Presence of FMR with impaired movement of leaflets during systole (type IIIb)—effective regurgitation orifice area greater than 20 mm^2^/regurgitant volume more than 30 ml/beat	Prolapse of anterior or posterior mitral valve leaflet (type II MR)
Left ventricular ejection fraction < 50% and/or left ventricular end-diastolic diameter ≥ 55 mm	Combined procedure with simultaneous aortic valve surgery
Tenting of the posterior and/or anterior mitral leaflet > 10 mm	Redo surgery of the mitral valve
	Previous coronary artery bypass graft (CABG) or valvular surgery (aortic, mitral, tricuspid)
	Degenerative mitral valve disease (e.g., extensive annulus calcification or leaflet/chordae fibrosis/restriction)
	Type I MR without leaflet tethering (i.e., atrial FMR)

### Preoperative assessment

As part of a mandatory preoperative screening, transthoracic echocardiogram (TTE), stress echocardiography, blood test, and 6-minute walk test were performed in all patients. QoL was also assessed in all patients using the SF-12 Health Survey. Cardiac magnetic resonance imaging (MRI) was performed in patients with no contraindications for a cardiac MRI in order to determine the ventricular functional reserve.

### Surgical technique

**Fig. 1:**
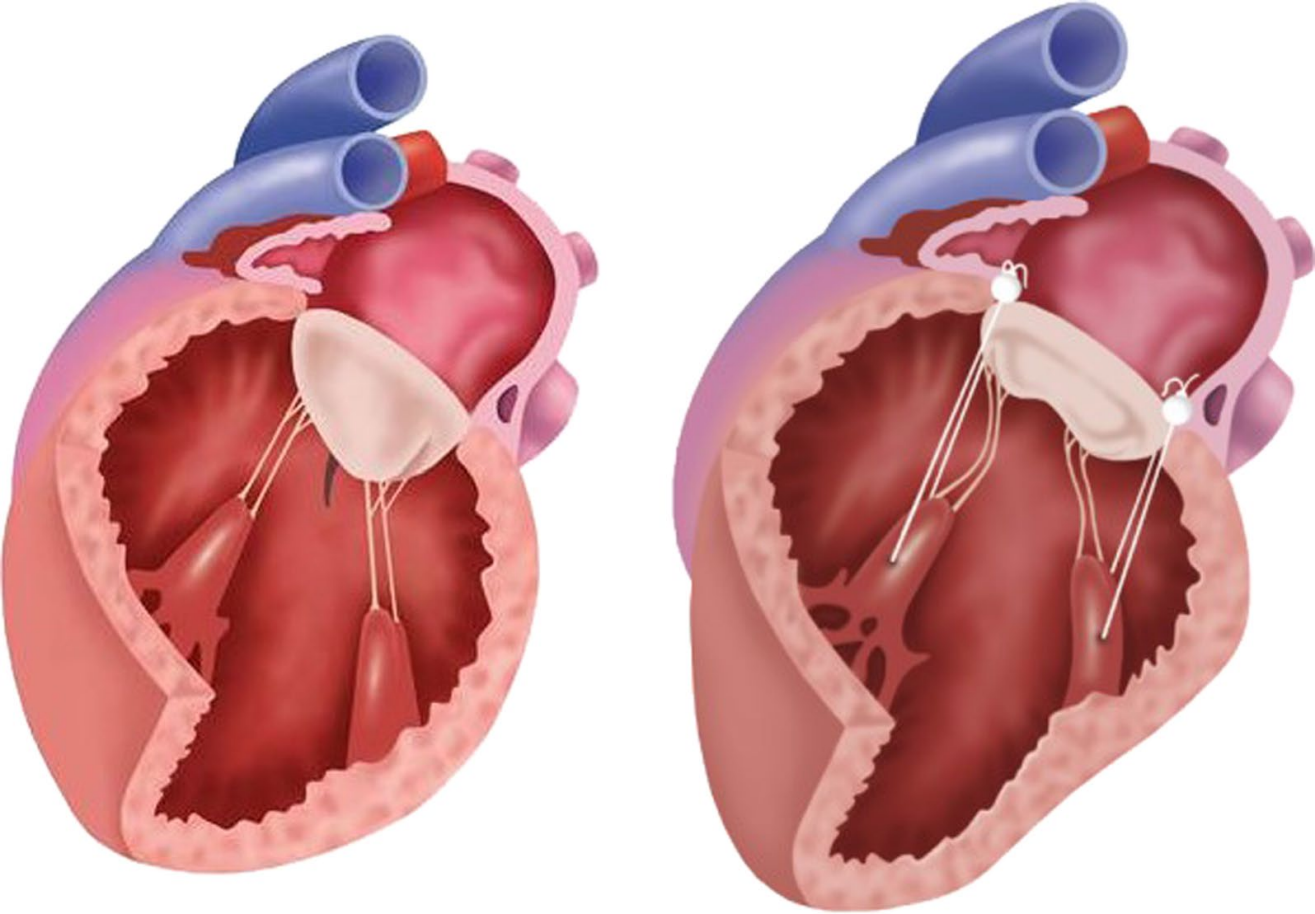
Standardized relocation of both papillary muscles using 2 pledgeted Polytetrafluoroethylene sutures

The technique of subannular repair by relocation of both papillary muscles has been described in detail previously (Fig. 1) [16]. The Carpentier-McCarthy-Adams IMR ETlogix annuloplasty ring (Edwards Lifesciences, Irvine, CA, USA) with a reduced antero-posterior diameter was used for a true-sized annuloplasty in all study patients. Briefly, double-armed pledgeted 3–0 Polytetrafluoroethylene sutures are passed through the trunks of both papillary muscles (i.e., anterolateral and posteromedial) in a U-formed fashion. Both suture ends are subsequently passed through the posterior mitral valve annulus from the ventricular to atrial side. Further, both sutures are retrieved outside the chest and secured for later use after placement of the annuloplasty ring. Next, the annuloplasty ring is being lowered down on the native mitral annulus while both sutures are kept outside the ring. After ring sutures are completely tied thereby securing the annuloplasty ring, both sutures are passed through the posterior aspect of the annuloplasty ring. In the next step of procedure, ”physiological” LV geometry is mimicked by filling the LV with a cold saline and thereby inducing a maximally possible tenting of mitral leaflets. While the LV is maximally filled, stepwise traction is applied to both sutures until leaflet tethering disappears and a mild “pseudoprolapse” of anterior mitral leaflet occurs. Both sutures are tightly knotted while keeping this traction and the knots are additionally secured with hemoclips. This maneuver allows for a very controlled realignment of both papillary muscles, reaching an appropriate distance between papillary muscle tips and mitral annular plane [16].

### Objectives

The primary objective of REFORM-MR is to evaluate the clinical outcomes of the standardized subannular repair in combination with annuloplasty, focusing on the recurrence of FMR (Grade 2 or more) in patients with type IIIb FMR at 2-year follow-up after the surgery (Table 2). Secondary objectives are the identification of echocardiographic predictors of recurrent MR in patients undergoing subannular repair. This includes the investigation of the underlying pathology of mitral valve and the analysis of the common tenting parameters that could serve as potential prognostic biomarkers. In addition, MACCE, mitral valve re-interventions, device therapy for advanced heart failure, mortality, re-hospitalization for heart failure, and QoL of patients with FMR before and after the surgery are assessed.

**Table 2:** REFORM-MR registry objectives

Primary objective	Freedom from recurrent FMR (> Grade 2) in patients with type IIIb FMR with reduced systolic ventricle function at 2-years follow-up after subannular repair by realignment of papillary muscles combined with annuloplasty
Secondary objectives	Assessment of common tenting parameters (tenting height, coaptation length, tenting area, posterior mitral leaflet and anterior mitral leaflet angles) via detailed echocardiographic examination and their correlation with the following clinical outcomes 6 months, 1 year, and 2 years after subannular repair combined with annuloplasty:
	Adverse cardiac events (MACCE)
	Mitral valve re-intervention
	Device therapy in advanced heart failure (Left ventricular assist device/Heart transplantation)
	Cardiac mortality
	Re-hospitalization due to heart failure
	QoL assessment of patients with FMR before and after subannular repair using SF-12 Health Survey
	Development of surgical strategies to improve long-term outcomes in patients with high-grade LV dilatation, including:
	Recurrent heart failure
	Long-term survival
	Ventricular function

### Data collection

The clinical outcome data collected are based on the site’s standards of care for MVr. The collected data include medical history, laboratory results and symptoms, TTE, and QoL measures, among others (Table 3). Data are captured by an electronic case report form (eCRF; Software for Trials Europe GmbH, Berlin, Germany) by either a study nurse or physician, and are checked automatically for plausibility and completeness. Digital Imaging and Communication in Medicine (DICOM) files of the echocardiograms are collected for analysis by the Echo Core Laboratory to ensure unbiased and consistent analysis of the echocardiographic data.

**Table 3:** Data collection schedule

	Preoperative parameters	Surgery	Discharge	6 months	Year 1	Year 2	Registry exit	Adverse events
Signed informed consent	X							
Inclusion/exclusion criteria	X							
Baseline characteristics	X							
Demographics and vital signs	X							
Medical history^a^	X							
Echo valve pathology	X							
Laboratory/Symptoms	X			X	X	X		
MRI	X				X	X		
Access (Operative data)		X						
Discharge data			X					
Echocardiogram (TTE)	X		X	X	X	X		
Echo form			X	X	X	X		
6 Minute Walk Test	X				X	X		
QoL questionnaire (SF-12)	X			X	X	X		
Outcome questions^b^				X	X	X		
Expiration (death form)			X	X	X	X		
Device and Patient success			X					
Registry exit							X	
Adverse events				X	X	X		X

*MRI* magnetic resonance imaging, *QoL* quality of life, *SF-12* short form-12, *TTE* transthoracic echocardiogram

^a^Includes current and previous cardiovascular and non‐cardiovascular conditions

^b^Includes occurrence of cardiovascular complications, site complications, and re-interventions since the last visit

### Monitoring

Physicians/surgeons and study personnel are required to make themselves familiar with the registry protocol, eCRF, requirements, and procedures. Approximately 20% of sites’ entries are selected at random and monitored after the completion of patient documentation, including follow-up. Source data verification is performed for all patients in these selected centers.

### Statistical analysis

Based on the expected recurrence of FMR between 10 and 50%, a sample of 100 patients will arrive at a 95% confidence interval (CI) of ± 8.98% for 30% FMR recurrence. The CI for 20% would be ± 7.84% and ± 5.88% for 10% FMR recurrence.

Statistical analyses are performed for the total study population. Continuous variables are presented as mean ± standard deviation (SD) or as median with interquartile range (IQR), and categorical variables (e.g., gender) are reported as frequencies and percentages. The Kolmogorov–Smirnov test is used to test for normal distribution. Comparisons are performed using Student’s t-test or Mann–Whitney U test for continuous variables and the Chi-Square or Fisher exact test for categorical variables. Linearized rates and actuarial probability statistics may be used where appropriate for adverse event reporting. Kaplan–Meier analyses is performed for survival and safety outcomes. All statistical analyses are performed using IBM SPSS Statistics version 24 (IBM, Armonk, New York). A *p*-value of < 0.05 is considered statistically significant.

## Results

As of May 2021, a total of 97 patients were enrolled across five sites in Germany.

## Discussion

Recurrent MR is common in patients undergoing an isolated annuloplasty for FMR, even in initially successful cases [10]. Although isolated annuloplasty is still considered as a gold standard for treating FMR, this procedure only corrects the mitral annulus without addressing the underlying pathophysiological mechanism of FMR. Liel-Cohen et al. initially highlighted the importance of papillary muscle tip-to-annulus distance in promoting mitral leaflet tethering [17]. Therefore, it seems quite logical to combine novel subannular repair manoeuvres with a ring annuloplasty for a pathophysiology-oriented management of FMR patients.

### Available data

Previous studies have demonstrated the benefits of adding various subannular manoeuvres to annuloplasty, including a reduced risk of MR recurrence, decreased leaflet tethering, reversed LV remodeling, and improved mid-term cardiac outcomes [18–20]. Since type IIIb FMR results from gradually increasing distance between the mitral annular plane and the tips of papillary muscles, applying papillary muscle interventions might prevent or at least delay the recurrence of MR by suppressing further apico-lateral papillary displacement. Data from meta-analyses suggest that a combination of papillary muscle techniques and annuloplasty is associated with significantly lower MR recurrence in FMR with restricted systolic leaflet motion when compared to annuloplasty alone [15, 21]**.** Fattouch and colleagues reported decreased mitral leaflet tethering and reduced rates of recurrent FMR and adverse cardiac events after additional papillary relocation as compared to an isolated annuloplasty [22]. Recently, Harmel et al. [23] published results of papillary muscle repositioning technique in combination with annuloplasty and similarly found a significantly lower recurrence rate of FMR at 1-year follow-up as well as decreased residual leaflet tenting after papillary muscle relocation vs. annuloplasty alone. Furthermore, papillary muscle relocation/repositioning has been shown to be more effective at preventing MR recurrence than papillary muscle approximation [21].

### Need for additional evidence

Despite the growing evidence suggesting that papillary muscles relocation is associated with better postoperative outcomes and longer overall survival, there is still no consensus regarding the most appropriate surgical strategy for subannular repair. Most reported data are from small, single-center, retrospective or observational studies [12, 22, 24]. Therefore, we designed a multicenter single-arm registry to further evaluate the combination of papillary muscle repositioning with annuloplasty for repairing type IIIb FMR. In addition, a better understanding of the tenting parameters and the underlying pathological mechanisms of recurrent MR will enable physicians to determine an individualised surgical strategy.

### Critical appraisal of the methodology

The REFORM-MR is a prospective, non-randomized single-arm registry conducted in multiple centres in Germany. The multicenter setting definitely increases the applicability of findings but might limit the generalizability of the results across other countries. Because of the multicentre design, an Echo CoreLab has been established to ensure unbiased and consistent analysis of the collected clinical data. In addition, Echo CoreLab can reduce variability of imaging data and ensure the validity of the results. Based on the recurrence rate of FMR, the sample size of 100 patients was determined and deemed sufficient for an accurate estimate of the predictive variables.

### Limitations

This registry is a non-randomized trial and has, therefore, a potential for confounding and bias in the analysis with a limited ability for adjustment. However, systematic data on patients’ outcomes after subannular MVr is scarce. Therefore, a prospective investigation of the preoperative parameters and postoperative outcomes in patients undergoing subannular MVr is valuable and may enable a wider use of this novel surgical approach in the clinical practice.

The lack of control group is another limitation of our study. Given the fact that the key point of this registry was implementation of standardized subannular repair technique in a multicenter setting and, therefore, included proctored cases as well as institutional learning curve, the general consensus was to start with a single-armed prospective registry with the focus on implementation of standardized subannular repair.

Finally, this manuscript is a protocol publication and describes the background and design of REFORM-MR registry only. However, taking into account that this registry represents the first multicenter initiative on a standardized subannular repair in type IIIb MR, we strongly believe that even a protocol paper has an apparent scientific value for the cardiac surgical community. It aims to further stimulate multicenter networking to create a solid evidence basis for pathophysiology-oriented repair in FMR and to encourage ongoing surgical research in this important area.

## Conclusion

The ultimate goal of REFORM-RM is to evaluate a standardized, reproducible, and effective papillary muscle relocation technique for type IIIb FMR in a multicenter setting. Thus, the results of this registry will provide supportive evidence of the efficacy of papillary muscle relocation in type IIIb FMR and in-depth information on the impact of tenting parameters on the outcomes of the subannular MVr. Together, these findings will help improve postoperative outcomes of FMR patients, reduce the risk of re-hospitalization, and potentially improve their long-term survival.

## Data Availability

Not applicable.

## Abbreviations

DICOM: Digital Imaging and Communication in Medicine
ECG: Electrocardiogram
eCRF: Electronic case report form
FMR: Functional mitral regurgitation
IQR: Interquartile range
LV: Left ventricle
MACCE: Major Adverse Cardiac and Cerebrovascular Events
MR: Mitral regurgitation
MVr: Mitral valve repair
SD: Standard deviation
QoL: Quality of life
